# Effectiveness of Aerobic Training for Adverse Symptoms Related to Chemotherapy During Treatment: Protocol for a Randomized Controlled Trial With Cost-Effectiveness Assessment

**DOI:** 10.2196/60828

**Published:** 2024-08-20

**Authors:** William de Lima Selles, Gisela Cristiane Miyamoto, Elinaldo da Conceição Santos, Cibelle Regina Lima Carmo, Giovanni Marini Moura, Giovanna Marques Frascoli Santos, Giovanna dos Santos Lopes, Diego Wisnieski Silva, Leticia Jeremias Ferreira Pereira, Adriana Claudia Lunardi

**Affiliations:** 1 Master's and Doctorate Program Universidade Cidade de São Paulo Sao Paulo Brazil; 2 Department of Biological and Health Sciences Universidade Federal do Amapá Macapá Brazil; 3 Department of Physical Therapy University of Sao Paulo Sao Paulo Brazil

**Keywords:** cancer, exercise, chemotherapy, physical therapy, cost benefit analyses, economic evaluation

## Abstract

**Background:**

One strategy to prevent adverse effects resulting from chemotherapy treatment is to perform physical exercises during treatment. However, there is still no consensus on the best type and intensity of exercise, nor when it should be started. Most studies have been carried out in patients with breast cancer, usually a few weeks after starting chemotherapy, on an outpatient basis 2 to 3 times a week. The main differences in our study are that we carried out physical training in hospitalized patients undergoing a cycle of chemotherapy for cancer treatment and that this training was carried out 5 times a week and was not restricted to a specific type of cancer.

**Objective:**

We aimed to evaluate the effects of aerobic training on symptoms related to chemotherapy (nausea, vomiting, asthenia, and sensation of weakness), fatigue, mobility, clinical complications, and length of hospital stay of patients during the drug treatment cycle. We also evaluated patient satisfaction with the proposed intervention, the adverse effects of aerobics training, and the cost-effectiveness of this intervention.

**Methods:**

This is a controlled and randomized trial with blinded evaluation that will include 94 hospitalized patients with cancer for 1 or more cycles of chemotherapy. The intervention group will perform aerobic training during a cycle of chemotherapy. The control group will receive a booklet with guidelines for staying active during the hospitalization period. The groups will be compared using a linear mixed model for fatigue, mobility, and chemotherapy-related symptoms before and after the intervention. The length of hospital stay will also be compared between groups using Kaplan-Meier survival analysis. The incidence of complications will be compared using the *χ*^2^ test. Cost-effectiveness and cost-utility analyses will be performed for the impact of exercise and quality-adjusted life years with the EQ-5D-3L-21 quality of life trials. The implementation variables (acceptability, suitability, and feasibility) will be evaluated by frequencies.

**Results:**

The clinical trial registration was approved in March 2023. Recruitment and data collection for the trial are ongoing, and the results of this study are likely to be published in late 2025.

**Conclusions:**

Chemotherapy has side effects that negatively impact the quality of life of patients with cancer. Aerobic exercise can reduce these side effects in a simple and inexpensive way. The field of work of physical therapists could be expanded to oncology if the intervention works.

**Trial Registration:**

Registro Brasileiro de Ensaios Clínicos RBR-6b4zwx3; https://tinyurl.com/39c4c7wz

**International Registered Report Identifier (IRRID):**

DERR1-10.2196/60828

## Introduction

Cancer is already the biggest public health problem in the world. In Brazil, the estimate for the 2023-2025 period is that there will be 704,000 new cases of cancer per year (excluding cases of nonmelanoma skin cancer) [1]. One of the main forms of treatment for cancer is chemotherapy, which consists of a systemic treatment in which a drug is used to interfere with cell proliferation, targeting its DNA or RNA and its metabolism [2]. The costs of cancer treatment in the United States already reach US $158 billion per year [3], and in Brazil, they exceed R $3.5 billion (US $700 million) [4]. The application of these resources requires health care staff, medications used directly and indirectly in treatment, equipment, and hospitalization days.

Treatment with chemotherapy involves a series of side effects, including nausea, vomiting, diarrhea, and febrile neutropenia, which are treated with supplementary medications. These adverse effects have a major impact on patients’ lives, as well as on adherence and compliance with the recommended dose of treatment, and they are also associated with an increase in the rate of hospitalization. At least one-third of women undergoing adjuvant chemotherapy treatment for breast cancer are unable to complete the prescribed dose of treatment due to adverse effects, thus compromising the prognosis of the disease [5].

Physical exercise in patients with cancer during chemotherapy has been tested in randomized trials [5-11] and appears to improve physical function, fatigue, symptom burden, and the quality of life of patients, in addition to allowing the nonreduction of chemotherapy doses. However, most studies have been carried out on an outpatient basis, involved patients with breast cancer, used 3 weekly exercise sessions, and evaluated fatigue as the primary outcome. There are still no clinical trials testing the effect of exercise in a hospital environment with daily sessions (Monday to Friday) carried out during the chemotherapy cycle.

Therefore, our objective is to evaluate the effects of aerobic training on chemotherapy-related symptoms (nausea, vomiting, asthenia, and sensation of weakness), fatigue, mobility, clinical complications, and length of hospital stay in patients during the drug treatment cycle. Furthermore, we also aim to evaluate the adverse effects of aerobic training, patient satisfaction with the proposed intervention, its implementation, and its cost-effectiveness.

Our hypothesis is that aerobic training will decrease chemotherapy-related symptoms, fatigue, clinical complications, and length of hospital stay, in addition to not increasing mobility. In addition, we hypothesize that the proposed intervention will be cost-effective from the point of view of the patient and society due to the preservation of quality of life and lower health costs related to the treatment of adverse effects from chemotherapy.

## Methods

### Design

This is the protocol for a randomized controlled trial with 2 parallel groups, 1:1 allocation, and blind evaluation that will be conducted in the oncology ward of the Instituto de Assistência Médica ao Servidor State Public, São Paulo-SP, Brazil, in accord with the CONSORT (Consolidated Standards of Reporting Trials) checklist [12]. The trial has been registered on the Registro Brasileiro de Ensaios Clínicos (RBR-6b4zwx3).

### Ethical Considerations

This study protocol was approved by the research ethics committees of the Universidade Cidade de São Paulo and Instituto de Assistência Médica ao Servidor (50436221.7.3001.5463). All participants will provide written informed consent before participating in this study. This will be collected and stored securely by one of the clinical data collection team members.

### Participants

Patients older than 18 years diagnosed with cancer and hospitalized in the ward to undergo at least 1 cycle of chemotherapy will be eligible for this study. Patients will be included in the study if they do not have musculoskeletal, cognitive, or clinical limitations that interfere with the proposed assessments and interventions, such as heart disease (eg, moderate to severe aortic or pulmonary stenosis, decompensated heart failure, advanced cardiac arrhythmias, myocarditis, and unstable coronary insufficiency), lung diseases (eg, chronic obstructive pulmonary disease or decompensated asthma), diabetes mellitus, and decompensated systemic arterial hypertension with serum hemoglobin <8g/dL or hematocrit <25% or platelets <30,000 mm [5,13-15].

Patients who require transfer to the intensive care unit, or for whom chemotherapy treatment has been contraindicated or discontinued for any reason, will be excluded from the study.

### Procedures

During the protocol period, all patients admitted to the oncology ward who are identified and meet the eligibility criteria will be invited to participate in the study by the evaluating physiotherapist. These eligible patients will be informed about the objectives of the study and invited to sign the informed consent form. Next, the patients will be evaluated regarding anthropometric and clinical characteristics, fatigue, and mobility, as well as the occurrence, frequency, intensity, and discomfort of the main adverse effects of chemotherapy. After the initial assessment, patients will be randomized into an intervention group, which will perform aerobic exercise sessions, and a control group, which will receive a booklet with guidelines (the same booklet will be given to the patients in the intervention group). The next day, the intervention will begin. Aerobic training will be carried out from Monday to Friday while the patients are hospitalized for the chemotherapy cycle in the oncology ward. Before carrying out the day’s aerobic exercise session, the patients will be asked if they feel any type of discomfort that is related to the previous day’s training (for example, muscle or joint pain in the lower limbs). If for any reason chemotherapy is interrupted for a patient, the exercises will also be interrupted, and as soon as chemotherapy treatment is resumed, the exercise will also be resumed. The booklet will be given to the patients and explained by a trained physiotherapist. Patients will be re-evaluated on the day following the last day of the chemotherapy cycle regarding the occurrence, frequency, intensity, and discomfort of adverse effects of the treatment, as well as fatigue, mobility, and satisfaction with the proposed intervention.

**Figure 1:**
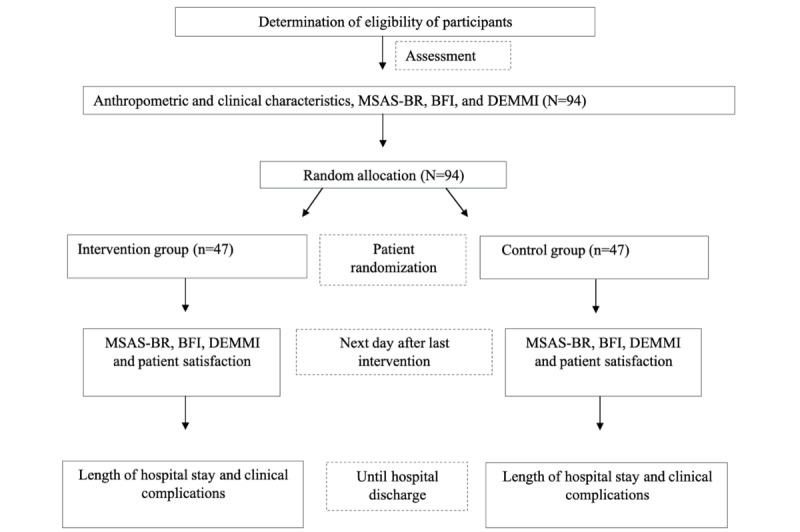
Clinical trial flowchart. BFI: Brief Fatigue Inventory; DEMMI: De Morton Mobility Index; MSAS-BR: Memorial Symptom Assessment Scale, Brazilian version.

After the study intervention period, patients who need it will receive routine physiotherapeutic care provided by the physiotherapy team at the hospital. The incidence of clinical complications from chemotherapy and the length of hospital stay will be assessed as outcomes until hospital discharge. All data will be collected by a blinded assessor. Additionally, data will be coded and entered into a Microsoft Excel spreadsheet and will be double-checked before analysis. A visual representation of the study design is presented in Figure 1.

### Randomization

Immediately after baseline assessments and before starting the intervention, patients will be randomly allocated to 1 of 2 groups: intervention or control. The allocation will be conducted by a researcher who will not be involved in the selection or evaluation of patients or in the intervention; it will follow a randomization schedule generated by a website [16] with a 1:1 allocation rate. The rooms in the ward accommodate 2 to 4 patients (beds); therefore, randomization will be carried out by group or cluster [17]. The rooms will be randomized, because although the intervention is directed to one patient, it can affect another patient in the same room, contaminating those who should not receive the intervention and reducing the estimated result [17]. The researcher responsible for randomization will place a written document inside a sealed opaque envelope indicating which group (intervention or control) each room belongs to in order to ensure the confidential allocation of patients to the blinded evaluator [18]. Before starting the intervention, the physiotherapist responsible for the intervention will open the envelope in front of the patient and disclose which group the room the patient is in corresponds to.

Due to the nature of the study, it will not be possible to blind the physiotherapist responsible for the intervention or the patients. However, the evaluator will be blinded to the groups (intervention and control). After the intervention and assessments, the physiotherapist responsible for the intervention will ask the blinded evaluator to which group (intervention or control) he believes each patient belonged to evaluate the evaluator’s blinding.

### Assessments

First of all, a standardized printed form will be used that will include personal data (name, age, sex, place of birth, contact telephone number, education, monthly family income, marital status, and occupation) and anthropometric data (height and weight, measured as recommended by the National Institute of Health, Heart, Lung, and Blood) [19]; diagnosis of current illness; preexisting illnesses; current or past smoking history; history of alcohol consumption and medications used; previous chemotherapy or radiotherapy; whether the current chemotherapy is neoadjuvant, adjuvant, treatment of choice, or palliative; and whether the patient is or was a practitioner of physical activity and how often they performed such activity.

The primary outcome will be assessed via frequency and intensity. Discomfort caused by the main adverse symptoms of chemotherapy (such as pain, nausea, and dry mouth) will be assessed using the Memorial Symptom Assessment Scale, Brazilian version (MSAS-BR) [20]. This scale consists of 32 symptoms that are scored by the patient according to their frequency and intensity (1-4 points, with 1 being less intense and frequent) and the discomfort (0-4, zero being no discomfort) that this symptom caused the previous week. This scale gives us a final index, which consists of the average of the 3 domains (frequency, severity, and bother) and all items, in addition to 4 subscales: Psych, which assesses psychological symptoms (with six items); Phys H, which assesses the most prevalent physical symptoms (with 12 items); Phys L, which assesses the less prevalent physical symptoms (with 14 items); and the Global Distress Index, which measures 4 psychological and 6 physical symptoms [20,21]. This scale will be applied in the initial and final assessments by an evaluator blind to which group the patient belongs.

Other outcomes will be assessed, such as fatigue, level of patient mobility, length of hospital stay, and clinical complications. Fatigue will be assessed using the Brief Fatigue Inventory, Brazilian version. This inventory consists of 9 items that are rated from 0 to 10 on a visual numeric scale. The first question is a dichotomous question about whether the patient has felt tired or fatigued in the last 7 days. Three items refer to the severity of fatigue at the time of assessment, the usual fatigue in the last 24 hours, and the worst fatigue in the last 24 hours, where 0 represents no fatigue and 10 “the worst fatigue imaginable.” The other 6 items refer to how fatigue interfered with different aspects of the patient’s life in the last 24 hours; these items are “general activities,” “mood,” “ability to walk,” “work” (including work outside and at home and daily tasks), “relationships with other people,” and “enjoying life,” where 0 means that it does not interfere and 10 means that it interferes completely [22,23]. This inventory will be applied in the initial and final assessments by an evaluator blind to which group the patient belongs.

The level of mobility will be assessed using the De Morton Mobility Index. This scale is made up of 15 activities, where 11 are classified as 0 or 1 (incapable or capable, respectively) and 4 are classified as 0, 1, or 2 (incapable, partial, and capable, respectively). Of the 15 activities, 3 are performed in bed, 3 in a chair, 4 involve static balance, 2 involve walking, and 3 involve dynamic balance. The scale was translated and validated in Brazilian Portuguese [24,25] and ranges from a score of 0, which represents low mobility, to 100, which represents high mobility. This index will be applied by an evaluator who is blind to which group the patient belongs and will be applied in the initial and final assessments.

In addition, the total number of days of hospital stay, possible clinical complications (cardiac, pulmonary, infectious, or neurological) arising from chemotherapy treatment, and the need for transfer to the intensive care unit will be accessed through the medical records of the patient.

The adverse effects of aerobic training, such as muscle pain, will be collected in the form of a question, developed by the researchers themselves, that will be answered every day before starting a new aerobic exercise session. In the same way, the patients’ subjective perception of satisfaction and discomfort in relation to the proposed protocol will be assessed through 2 questions developed by the researchers themselves that will be answered on the day of the patients’ final assessment. The patients’ responses to the questions will be on a scale of 0 to 10. In the question about satisfaction with the treatment, 0 will indicate “complete dissatisfaction” and 10 “maximum satisfaction.” When the question is about the discomfort of the treatment, 0 will indicate “no discomfort” and 10 will indicate “maximum discomfort.”

At the end of the treatment, the acceptability, suitability, and feasibility outcomes will be assessed using the Weiner scale [26], which was translated and adapted into Brazilian Portuguese [27]. The answer options for all questions are “completely disagree” (1 point), “disagree” (2 points), “neither agree nor disagree” (3 points), “agree” (4 points) and “completely agree” (5 points).

Acceptability is the perception among parties interested in implementing a given treatment that it is pleasant or satisfactory. It will be evaluated in terms of patient satisfaction in the intervention group according to their responses to the following statements: (1) “Physical exercise during chemotherapy treatment has my approval”; (2) “Physical exercise during chemotherapy treatment is attractive to me”; (3) “I enjoyed physical exercise during chemotherapy treatment”; and (4) “I recommend physical exercise during chemotherapy treatment.”

As for suitability, it is the relevance or compatibility of the proposed innovation (ie, treatment) for a given clinical practice environment. It will be evaluated by the following statements addressed to health professionals who care for patients included in this study, such as doctors, nurses, and physiotherapists: (1) “Physical exercise during chemotherapy treatment seems appropriate”; (2) “Physical exercise during chemotherapy treatment seems adequate”; (3) “Physical exercise during chemotherapy treatment seems applicable”; and (4) “Physical exercise during chemotherapy treatment seems to be a good option.”

Feasibility is defined as the extent to which a new treatment can be implemented within a given environment. It will be evaluated by the following statements addressed to physiotherapists and patients included in this study: (1) “Physical exercise during chemotherapy treatment seems implementable”; (2) “Physical exercise during chemotherapy treatment seems possible to perform”; (3) “Physical exercise during chemotherapy treatment seems feasible”; and (4) “Physical exercise during chemotherapy treatment seems easy to use.”

Economic evaluation will be monitored during the hospitalization period [28]. The index year will refer to the year of data collection. The costs of the intervention used will be assumed through the estimated value of the instruments used (oximeter and cycle ergometer), as well as the booklets and the in-hospital physiotherapy sessions (using the reference fees from the Physiotherapy Council table). The assessment of health care costs corresponds to the individual’s costs to the health care system and the costs of lost productivity due to total days of hospitalization. This assessment will be carried out using cost information obtained at the hospital. The cost-effectiveness analysis will be carried out by evaluating the effects of the intervention using the MSAS-BR-16 questionnaire, while the cost-utility analysis will be measured by quality-adjusted life years (QALYs) using the quality-of-life questionnaire EQ-5D-3L [29]. Sensitivity analysis will test uncertainty in key parameters, such as selection of cost weights and statistical variation in quality-of-life scores.

### Interventions

The patients allocated to the intervention group will participate in an aerobic training protocol on a cycle ergometer during the period in which they are hospitalized and undergoing the chemotherapy cycle. The training will be carried out with daily sessions for 4 days lasting 40 minutes as long as the chemotherapy cycle continues. Exercise on the cycle ergometer will begin with a 5-minute warm-up and will progress to 30 minutes of exercise at a speed corresponding to 40%-60% of the heart rate reserve calculated by the Karvonen formula [30]: (220 – age) – (heart rate of rest × % intensity) + resting heart rate. The exercise will end with 5 minutes of recovery. The objective will be to maintain the patient at a score of 12 for the perception of dyspnea and physical fatigue, as measured by the Borg scale [31] (12 indicates that the patient’s dyspnea and physical fatigue is above “relatively easy” and below “slightly tiring”), during exercise on the cycle ergometer. The Borg scale presents a range of scores from 6 (no effort) to 20 (maximum effort). Therefore, if the patient is considered fit by the physiotherapist, the speed on the cycle ergometer will be gradually increased until the patient reaches 60% of the heart rate reserve. We will record heart rate and peripheral oxygen saturation (Palmsat 2500 pulse oximeter; Nonin), dyspnea and physical fatigue (Borg scale) [28], and blood pressure (Premium Aneroid Sphygmomanometer and Littmann Classic II Stethoscope) before, during, and after each physical training protocol session. The session may be interrupted at any time if the patient presents signs or symptoms such as dizziness, dyspnea, fatigue, or nausea. If the patient requires any emergency assistance during the period in which they are undergoing physical training supervised by the study physiotherapist, the same physiotherapist will contact the medical team on duty responsible for the patient in the oncology ward to provide the necessary assistance to stabilize the condition of the patient. To assist in blinding the evaluator, a booklet titled “Why do I need to move when I am hospitalized” [32] will be given to patients in both groups. The cycle ergometer will always be removed from the patient’s room after the training session to ensure blind evaluation.

The patients allocated in the control group will receive the same booklet (“Why do I need to move when I’m hospitalized”), which uses illustrations and short texts to encourage patients to spend more time in their chair, walk, and go up and down stairs (with a companion) [32]. This booklet will be delivered and explained by a trained physiotherapist and the participant will keep it for any questions and consultations.

### Statistical Design

The calculation to determine the sample size was carried out based on a previous randomized clinical trial that also compared the effect of aerobic training compared to usual care on the deleterious effects of chemotherapy in patients with breast cancer [11]. Our study was designed to detect a difference of 2.7 (SD 4) points in the physical fatigue score, in addition to a difference of 6.2 (SD 7) episodes of vomiting or nausea between the groups. A power of 80%, an α of 5%, and a loss to follow-up of 15% of the sample were considered. A sample of 94 patients (47 patients in each group) was estimated.

After the data collection, all analyses will be conducted following intention-to-treat principles and will be performed by a researcher who will not be involved in the study assessments and intervention.

For analyzing the effect of the intervention, after a descriptive analysis and the Shapiro-Wilk normality test with a visual analysis, the groups will be compared using a linear mixed model for fatigue, mobility, and chemotherapy-related symptoms before and after the intervention. The length of hospital stay will also be compared between groups using Kaplan-Meier survival analysis. The *χ*^2^ test will be used to compare the clinical complication rates between groups. Descriptive analysis will be used for recruitment, completion, and adherence rates and potential adverse events. The significance level will be set at 5%. For all these analyses, SPSS (version 21.0; IBM Corp) will be used.

For economic evaluation, the analysis will be performed using chemotherapy-related symptoms, fatigue, and mobility as outcomes, and the cost-utility analysis will use QALYs. Missing data will be handled using multiple imputation by chained equations. Ten complete data sets will be created (efficiency loss <5%). Pooled estimates will be calculated according to the Rubin rules [33]. Average cost differences between groups will be calculated for total and disaggregated costs. Incremental cost-effectiveness and cost-utility rates will be calculated by dividing the difference in total costs by the difference in effects. Uncertainty surrounding cost differences and incremental cost-effectiveness and cost-utility ratios will be estimated using corrected and accelerated bootstrap techniques (5000 replications). The latter will be presented graphically in cost-effectiveness plans [34]. Cost-effectiveness acceptability curves will be estimated to indicate the probability of interventions being cost-effective compared to each other at different willingness-to-pay values [35]. Sensitivity analyses will be performed to assess the robustness of the results. The first sensitivity analysis will be performed from a health care perspective, and the second sensitivity analysis will be performed per protocol. The economic evaluation will be carried out using Stata (version 14; StataCorp).

## Results

In March 2023, the clinical trial registration was approved. Recruitment and data collection for the trial is ongoing, and the results of this study are likely to be published in late 2025.

## Discussion

### Anticipated Findings

To our knowledge, this will be the first controlled trial with an economic analysis that will be carried out to test the acute effects of aerobic exercise in patients undergoing a cycle of chemotherapy. However, based on previous studies conducted in the outpatient phase of cancer treatment [36], we believe that aerobic training will be cost-effective and will lead to a lower incidence of adverse effects from chemotherapy treatment, less fatigue, maintenance of mobility, shorter hospital stays, and lower incidence of clinical complications when compared to the control group.

Despite the potential beneficial effects of physical exercise for patients with cancer, a decline in activity level has been reported in this population [37]. This decline appears to have an impact on quality of life and to lower performance in activities of daily living in women with breast cancer. A possible hypothesis for this decrease in physical exercise may be the relationship with psychosocial barriers in these patients [37].

Smith and colleagues [38] conducted a study that aimed to assess exercise behavior, barriers, facilitators, and motivators for exercise participation and the different exercise support needs of cancer survivors living in a rural Canadian community. They observed that the main barriers reported were cost, time, distance, transportation, and side effects. Still, the study described above was able to observe that patients reported that access to a gym, equipment, social support, transportation, and information on physical exercise were facilitators [38].

The Clinical Oncology Society of Australia and Exercise and Sport Science Australia encourage health care professionals who work with patients with cancer to seek to know more about the relationship between physical activity and cancer and understand how they must guide their patients to practice physical activity [39]. They also advise health professionals to refer patients to professionals who are more prepared and who better understand the practice of exercise in patients with cancer.

In the study by Park and colleagues [40], the authors analyzed the influence of doctors on their patients among 162 people who overcame early-stage breast and colorectal cancer and who completed primary and adjuvant treatment. Only 80.7% (n=130) of participants in the study remained until the end. Three groups were stratified: the first was the control group (n=59); the second group received exercise recommendations from oncologists (n=53); and the third group received physical activity recommendations from oncologists and an exercise motivation package (n=50). Participants were assessed at the beginning of the study and after 4 weeks [40].

The motivation package provided exercise DVDs, a pedometer, an exercise diary, and a 15-minute physical education session. In the recommendations, the oncologists said, “Studies have shown that engaging in moderate PA [physical activity] more than 150 minutes per week could significantly reduce the recurrence of breast and colorectal cancer. Therefore, it is highly recommended that breast and colorectal cancer survivors participate in at least 150 minutes of moderate-level PA and twice-weekly strengthening exercises” [40].

We have seen in the current literature the importance of understanding how much physical exercise can impact the treatment and quality of life of patients with cancer undergoing chemotherapy treatment. Studies have shown that incorporating adequate and supervised exercise can help combat fatigue, improve physical fitness, reduce anxiety and depression, and help maintain functionality. However, it is important that the exercise program is individualized, taking into account the stage of the disease, as well as the patient’s age, needs, and limitations. Supervision by specialized professionals is essential to ensure safety and effectiveness. In summary, physical exercise can be a valuable tool in managing symptoms and promoting well-being in patients with cancer, complementing conventional treatments and providing both physical and emotional improvements during the cancer treatment journey.

### Conclusion

Chemotherapy has side effects that negatively impact the quality of life of patients with cancer. Aerobic exercise can reduce these side effects in a simple and inexpensive way. Oncology could be a way to expand the field of work for physical therapists if the intervention works.

## Appendix

Multimedia Appendix 1 Peer review reports from the Pró-Reitoria de Pós-Graduação, Pesquisa e Extensão (PRPGPE) - Universidade Cidade de São Paulo (UNICID) - São Paulo Research Foundation (FAPESP) (Brazil).

## Abbreviations

CONSORT: Consolidated Standards of Reporting Trials
MSAS-BR: Memorial Symptom Assessment Scale
QALY: quality-adjusted life year

## Data Availability

The data obtained in this study will be available from the corresponding author (ACL) upon reasonable request.
