# Supplementary material for: Effectiveness of Aerobic Training for Adverse Symptoms Related to Chemotherapy During Treatment: Protocol for a Randomized Controlled Trial With Cost-Effectiveness Assessment
Source: JMIR Res Protoc. 2024 Aug 20;13:e60828. doi: 10.2196/60828 (PMC11372328; doi:10.2196/60828)
Supplement: Multimedia Appendix 1 [file resprot_v13i1e60828_app1.pdf]

FAPESP SAGe

VIEW DISPATCH

**Process**	2023/07735-8
**Line of Promotion**	Regular Programs / Research Grants / Research Project / Research Project - Regular - Continuous Flow
**Status**	In progress
**Duration**	01/02/2024 a 31/01/2026
**Beneficiary**	Adriana Claudia Lunardi
**Responsible**	Adriana Claudia Lunardi
**Link Institutional Process**	Dean of Postgraduate Studies, Research and Extension/PRPGPE/UNICID
**Title**	EFFECTIVENESS OF AEROBIC TRAINING ON ADVERSE SYMPTOMS RELATED TO CHEMOTHERAPY DURING TREATMENT: RANDOMIZED CONTROLLED TRIAL WITH ECONOMIC EVALUATION.

Initial Proposal Clearance Sheet - Research Project - Regular

Results

Denied

Dates of dispatch

Issued on : 16/10/2023

Consolidated Budget

Benefits	Requested		Dispatch	
	Value (R$)	Value (US$)	Value (R$)	Value (US$)
Capital				
Permanent Material	12.586,08	0,00	0,00	0,00
Costs				
Transportation costs	0,00	0,00	0,00	0,00
Daily rates	0,00	0,00	0,00	0,00
Consumables	0,00	0,00	0,00	0,00
Third-party services	0,00	5.760,00	0,00	0,00
Technical Reserve - Complementary Benefits	32.000,00	0,00	0,00	0,00
Technical Reserve - Direct Project Infrastructure Cost	6.294,31	0,00	0,00	0,00
Provision for Imports	0,00	864,00	0,00	0,00
Total	50.880,39	6.624,00	0,00	0,00

Scholarship Quotas

Requested				Dispatch			
Modality / Level	Workload	Duration (Months)	Quantity	Type / Level	Workload	Duration (Months)	Quantity

No quota requested.

Project Team

Team Members - Requested

Name	Function	BC Requested	Period requested
Adriana Claudia Lunardi	Researcher in Charge	Yes	01/08/2023 - 31/07/2025
Gisela Cristiane Miyamoto	Associate Researcher	-	01/08/2023 - 31/07/2025
Diego Wisnieski da Silva	Students without grants	-	01/08/2023 - 31/07/2025
Giovanni Marini Moura	Students without grants	-	01/08/2023 - 31/07/2025
William de Lima Selles	Students without grants	-	01/08/2023 - 31/07/2025

Execution data

**Start Date**	01/08/2023
**Duration**	24 month(s)
**End Date**	31/07/2025
**Resource allocation area**	Health
**Scientific Report (Quantity)**	1
**Scientific Report (Dates)**	01/01/1900
**Accountability (Quantity)**	1
**Accountability (Dates)**	01/01/1900
**Research Category**	B/T/PP

Observations / Transcriptions / Phrases

Remarks to the person in charge

We would like to inform you that your request for research assistance, included in the above-mentioned process, was analyzed by FAPESP's advisory board and was denied.

To find out the content of the order, please access the SAGe System (www.fapesp.br/sage), selecting the menu item My Processes>>Process Number and, under More Information, the option Order.

Please use the "Converse com a FAPESP" service exclusively at www.fapesp.br/converse for any queries or communications regarding this correspondence.

We remain at your disposal for future requests.

Sincerely,

Marcio de Castro Silva Filho
Scientific Director

Phrases for the person in charge

There are no associated phrases.

Transcript of Opinion for the Person in Charge

OPINION OF THE COORDINATING BODIES

The proposal was well evaluated by the advisory board. The applicant has regular scientific production with an impact and established international collaborations, as well as experience in training human resources.
In response to the diligence, the researcher filled in the budget request (total of R$50,880.39 + U$6,624.00) which was considered adequate, except for the request for publication aid (2 x 2,390.00) which is financed by the RT of the process.

However, no aid of any kind was requested for the development of the project. In the documents submitted, there is no justification or commitment that the project can be carried out and that it has the full infrastructure of equipment and supplies for its development.

ADVISORY OPINION

ANALYSIS OF THE PROPOSAL. The analysis of proposals submitted as Regular Research Grants includes the Evaluation of the Research Project, the Academic Record of the Researcher Responsible for the Project and the Budget. This form is made up of three sections referring to each of the parts, with a General Assessment of the Research Project (AGP) at the end.

I. About the proposed RESEARCH PROJECT

Criterion 1: Original, internationally competitive and well-presented research project. The proposal under analysis meets the criterion:

[X] Very [ ] Quite a lot [ ] Fairly [ ] Little [ ] Not at all

----

Please justify the above choice (The strength of the justification will be a decisive factor in FAPESP's analysis of the proposal):

Original, well-structured project with a relevant research question and clinical and socially relevant applicability.

Criterion 2: The research challenges are adequately formulated and situated in relation to the state of the art and the existing literature. The proposal under analysis meets this criterion:

[ ] Very [X] Quite a lot [ ] Fairly [ ] Little [ ] Not at all

----

Please justify the above choice (The strength of the justification will be a decisive factor in FAPESP's analysis of the proposal):

The project makes clear the state of the art and the gaps that the research aims to fill.

Criterion 3: The methodology is appropriate, well justified and sufficiently detailed. The proposal under analysis meets the criterion:

[X] Very [ ] Quite a lot [ ] Fairly [ ] Little [ ] Not at all

----

Please justify the above choice (The strength of the justification will be a decisive factor in FAPESP's analysis of the proposal):

Well-described methodology with robust and adequate statistical analysis.

Criterion 4: The results have the potential to significantly expand the frontier of knowledge in the area and, therefore, to have a relevant scientific impact, with the publication of papers with the potential to be cited by the community in the area's literature. The proposal under analysis meets this criterion:

[ ] Very [X] Quite a lot [ ] Fairly [ ] Little [ ] Not at all

----

Please justify the above choice (The strength of the justification will be a decisive factor in FAPESP's analysis of the proposal):

The project has a high chance of being published in impact journals and cited in the field.

Criterion 5: The research project can be carried out by the responsible researcher and his/her team within the timeframe set out in the proposal. The proposal under analysis meets the criterion:

[ ] Very [X] Quite a lot [ ] Fairly [ ] Little [ ] Not at all

----

Please justify the above choice (The strength of the justification will be a decisive factor in FAPESP's analysis of the proposal):

The work schedule presented is adequate

Criterion 6: The Data Management Plan describes the types of data produced by the project and how they are stored, preserved and shared. The proposal under analysis meets the criterion:

[ ] Very [ ] Quite a lot [X] Fairly [ ] Little [ ] Not at all

----

Please justify the above choice (The strength of the justification will be a decisive factor in FAPESP's analysis of the proposal):

The management plan can be improved with certain information, and I recommend the DMPTool platform for a more complete plan.

II. About the ACADEMIC BACKGROUND of the Researcher Responsible for the Project

----

Criterion 7: The scientific results published by the Principal Investigator in the research area of the proposed project have a relevant scientific impact. The proposal under analysis meets this criterion:

[ ] Very [X] Quite a lot [ ] Fairly [ ] Little [ ] Not at all

----

Please justify the above choice (The strength of the justification will be a decisive factor in FAPESP's analysis of the proposal):

She has 59 papers published in indexed journals, with an H-index of 15.

Criterion 8: The researcher demonstrates scientific leadership or, in the case of early career researchers, their leadership potential is evident. The proposal under analysis meets this criterion:

[ ] Very [X] Quite a lot [ ] Fairly [ ] Little [ ] Not at all

----

Please justify the above choice (The strength of the justification will be a decisive factor in FAPESP's analysis of the proposal):

He has led 3 projects with regular FAPESP funding

Criterion 9: The researcher has experience in training researchers that is compatible with the stage of the

career and institutional conditions. The proposal under analysis meets the criterion:
[ ] Very [X] Quite a lot [ ] Fairly [ ] Little [ ] Not at all

---

Please justify the above choice (The strength of the justification will be a decisive factor in FAPESP's analysis of the proposal):

He has already completed 13 master's degrees and 3 doctorates. He is currently supervising 3 master's degrees, 2 doctorates and one post-doctoral supervision.

Criterion 10: The researcher's academic record demonstrates international research experience after their doctorate or active participation in international research collaboration networks. The proposal under analysis meets the criterion:
[ ] Very [X] Quite a lot [ ] Fairly [ ] Little [ ] Not at all

---

Please justify the above choice (The strength of the justification will be a decisive factor in FAPESP's analysis of the proposal):

The researcher has been in partnership with Professor William Poncin of the Université Catholique de Louvain in Belgium since 2019. She is in the early stages of a partnership with Christina Ekegren, a researcher at Munish University in Melborne, Australia.

III. About the requested BUDGET

Evaluation 1: Are the equipment and permanent materials requested to carry out the project well justified in the proposal, given the existing infrastructure at the host institution and the ability of the requesting team to use them?

[X] Yes
[ ] No

In each case, give your opinion on the validity of the justifications presented, considering the need for the objectives of the research and the possible availability of similar equipment in the institution itself. (The "Equipment Park" document, which is mandatory for analysis, must present a list of equipment available in the institution).

There were no requests for permanent equipment

Assessment 2: The items of consumable material requested for the project are justified in the proposal.

[X] Yes
[ ] No

In each case, give your opinion on the validity of the justifications presented, taking into account their necessity for the objectives of the research.

there was no request for consumables

Evaluation 3: The third-party services requested to carry out the project are justified in the proposal and are only of a technical and occasional nature, as required by FAPESP rules.

[X] Yes
[ ] No

In each case, give your opinion on the validity of the justifications presented, taking into account their necessity for the objectives of the research.

there was no request for consumables

If necessary or appropriate, please suggest alternative budget figures. Important Note: Salaries of any kind, third-party services other than technical and occasional services, civil works, the purchase of publications, travel (except for field research), supplies and administrative services should be excluded from the budget. BUDGET REQUESTED:

#	Heading	Qty	Requested R$	US$	Qty	R$	US$
	**Recommended 1 Capital and Funding**		**32.000,00**	**0,00**		**32.000,00**	**0,00**
2	Technical Reserve						
3	Technical Reserve - Complementary Benefits	1	32.000,00	0,00	1	32.000,00	0,00
4	Technical Reserve - Direct Project Infrastructure Cost		0,00	0,00		0,00	0,00
5	Provision for Imports		0,00	0,00		0,00	0,00

**6 Grand total**	**32.000,00**	**0,00**	**32.000,00**	**0,00**

IV. GENERAL APPRAISAL OF THE PROPOSAL (AGP)

Strengths (in particular, point out the most original or innovative aspect of the proposed research project)

The strength of the proposal is that it provides more information and proposes an exercise protocol to be carried out during the chemotherapy cycle of hospitalized patients in order to reduce the adverse effects caused by the procedure. In short, the relevance and clinical applicability are the strongest points of the proposal, which is well outlined and has an appropriate methodology.

----

a) On the Research Project, as indicated in item I:

[ ] Project with poorly defined, excessive or incongruous objectives. [
] Project with excessively limited objectives.
[ ] Unoriginal design.
[ ] Poorly formulated research challenges.
[ ] Insignificant contribution to the field [ ] Inadequate
methodology.
[ ] Execution feasibility questionable. [ ]
Inadequate timeframe.
[ ] Inadequate and/or insufficient data management plan

----

b) About the Researcher in Charge, as indicated in item II:

[ ] Insufficient experience in the research area of the project, which could compromise its viability.
[ ] Scientific or technological production that does not attest to the significant performance of the research activity. [ ] Insufficient experience in training researchers
[ ] Little international experience.

----

c) On the proposed budget, as indicated in item III

[ ] Excessive cost in relation to the expected scientific or technological contribution or the likelihood of the project's success.
[ ] Equipment and Permanent Materials insufficiently justified. [ ]
Insufficiently justified items of consumables.
[ ] Third-party services insufficiently justified

----

d) Weaknesses (Other - justify):

Conclusion

The proposal consists of a highly relevant and well-designed project, a researcher with experience in leading projects and training human resources. There are no budgetary demands apart from the supplementary benefit.

Phrases for the Grant Agreement

There are no associated phrases.

Detailed Budget - Summary Tables

Permanent Material - National

Item	Description	Requested			Dispatch		
		Qty	Unit Value (R$)	Total Value (R$)	Qty	Unit Value (R$)	Total Value (R$)
1	Arm Leg Exerciser Mini Bike Cycle ergometer with Monitor	2	635,00	1.270,00	0	0,00	0,00
2	Ut100-MD portable pulse oximeter	2	2.570,00	5.140,00	0	0,00	0,00
2.1	Duracell Alkaline Battery Small AA - BT 16UN	10	68,80	688,00	0	0,00	0,00
3	Welch Allyn Ds44 Sphygmomanometer Kit + Double Stethoscope B...	2	699,99	1.399,98	0	0,00	0,00
3.1	Welchy Allyn Flexiport N10 Adult Cuff	1	249,90	249,90	0	0,00	0,00
4	Tablet Samsung Galaxy A7 Lite 64GB 8.7 Inch Android 11 ...	2	1.299,00	2.598,00	0	0,00	0,00
5	Deskjet Ink Advantage 2774 7FR22A Multifunction Printer,...	1	359,10	359,10	0	0,00	0,00
